# Selective internal radiation therapy of hepatic tumors: procedural implications of a patent hepatic falciform artery

**DOI:** 10.1186/2193-1801-3-595

**Published:** 2014-10-10

**Authors:** Juliane Schelhorn, Judith Ertle, Joerg F Schlaak, Stefan Mueller, Andreas Bockisch, Thomas Schlosser, Thomas Lauenstein

**Affiliations:** Department of Diagnostic and Interventional Radiology and Neuroradiology, University Hospital Essen, Hufelandstrasse 55, 45147 Essen, Germany; Department of Gastroenterology und Hepatology, University Hospital Essen, Hufelandstrasse 55, 45147 Essen, Germany; Clinic of Nuclear Medicine, University Hospital Essen, Hufelandstrasse 55, Essen, 45147 Germany

**Keywords:** Internal radiation therapy, Radioembolization, Hepatic falciform artery, Non-targeted irradiation

## Abstract

Selective internal radiation therapy (SIRT) using 90-yttrium is a local therapy for unresectable liver malignancies. Non-targeted 90-yttrium diversion via a patent hepatic falciform artery (HFA) is seen as risk for periprocedural complications. Therefore, this study aimed to evaluate the impact of a patent HFA on SIRT. 606 patients with SIRT between 2006 and 2012 were evaluated retrospectively. SIRT preparation was performed by digital subtraction angiography including ^99m^Tc-HSAM administration and subsequent SPECT/CT. Patients with an angiographically patent HFA were analyzed for procedural consequences and complications. 19 of 606 patients (3%) with an angiographically patent HFA were identified. Only 11 of these 19 patients received 90-yttrium in the hepatic vessel bed containing the HFA. Initial coil embolization of the HFA succeeded only in three of 11 patients. Out of the eight remaining patients four had no abdominal wall ^99m^Tc-HSAM accumulation. The other four patients presented with an abdominal wall ^99m^Tc-HSAM accumulation, for those a reattempt of HFA embolization was performed or ice packs were administered on the abdominal wall during SIRT. In summary, all patients tolerated SIRT well. A patent HFA should not be considered a SIRT contraindication. In patients with abdominal wall ^99m^Tc-HSAM accumulation HFA embolization or ice pack administration seems to prevent complications.

## Background

Selective internal radiation therapy (SIRT) using 90-yttrium microspheres is increasingly used as a treatment option for unresectable primary and secondary liver malignancies (Salem and Thurston 2006a, [b], [c]; Salem et al. 2005, 2007; Lewandowski et al. 2007; Powerski et al. 2012; Wybranski et al. 2009; Dudeck et al. 2010). Due to the mainly arterial blood supply of the tumor, but the dual arterial and portal-venous blood supply of the liver, the intraarterial application of the pure β-emitter 90-yttrium allows the administration of a high radiation dosage into the tumor with partially sparing the healthy liver tissue. To achieve the required therapeutic radiation dosage in the tumor, approximately 1.0 - 3.0 GBq have to be applied (Salem and Thurston 2006a; Wybranski et al. 2009; Grober et al. 2011). Respectively the non-targeted diversion of 90-yttrium microspheres to other organs may lead to severe complications such as radiation gastritis, gastrointestinal ulcers, pancreatitis, and pneumonitis (Salem and Thurston 2006b; Paprottka et al. 2012; Pech et al. 2009; Dudeck et al. 2011). Less dangerous, nevertheless compromising, self-limiting periumbilical radiation dermatitis and severe periumbilical pain due to non-targeted diversion of 90-yttrium microspheres to the anterior abdominal wall via a patent hepatic falciform artery (HFA) have been reported (Leong et al. 2009; Bhalani and Lewandowski 2011). Hence, the aim of this study was to evaluate the frequency and the impact of an angiographically patent HFA in SIRT and to assess strategies to reduce concomitant complications.

## Methods

### Patients

Retrospective analysis and use of data was approved by the local ethics committee (ethics committee of the University Essen). All 606 patients (464 male, 142 female, mean age 64.5 years) who underwent SIRT between October 2006 and December 2012 at our hospital were reevaluated with respect to an angiographically detectable patent HFA. All patients were not eligible for curative treatment due to their advanced tumor disease. The tumor entities were hepatocellular carcinoma (n = 476), cholangiocarcinoma (n = 14), and hepatic metastases (n = 116). Cirrhotic patients with Child Pugh score >7 points were not eligible for SIRT.

### Digital subtraction angiography (DSA)

DSA was performed on a biplanar DSA system (Philips Allura, Philips Healthcare, Best, The Netherlands; or Toshiba Infinix DP-i, Toshiba Medical Systems, Tokyo, Japan). Using Seldinger technique a 5 F vascular sheath (Avanti + Sheath Introducer, Cordis Europe, Waterloo, Belgium) and a 5 F guiding catheter (Sidewinder-1, Sindwinder-2 or Cobra-2; Terumo Europe, Leuven, Belgium) were inserted via a transfermoral access. Selective DSA of the celiac trunk and the superior mesenteric artery was performed with 15 ml of contrast agent (Xenetix, Guerbet, Roissy, France) at a flow rate of 5 ml/s by an automatic injector (Tyco Healthcare, Mansfield, MA, USA). Imaging parameters were 80 kV, 70 mAs, and 2 images/s. After visualization of the tumor extent an appropriate microcatheter (Rebar 0.27 inch, ev3 Europe SAS, Paris, France) position for the ^99m^Tc-HSAM (99 m-technetium labelled human serum albumin microspheres, ROTOP Pharmaka AG, Dresden, Germany) administration was defined for the right, left, or both hepatic lobes. If the gastroduodenal artery or the right gastric artery were identified near the intended injection site, they were occluded by permanent coil embolization using interlocking detachable or pushable coils (Boston Scientific, Natick, MA, USA or Cook Medical, Bloomington, IN, USA; microcatheters for coiling: Rebar 0.18 inch, ev3 Europe SAS, Paris, France or Renegade 21, Boston Scientific, Natick, MA, USA). A patent HFA in DSA was attempted to probe and coil embolize. Finally, a total of 150 MBq ^99m^Tc-HSAM was injected at the defined microcatheter positions.

### Single-photon emission computed tomography/computed tomography (SPECT/CT)

SPECT/CT images were acquired using a Symbia SPECT/CT system (Siemens Healthcare, Erlangen, Germany) 60 minutes after ^99m^Tc-HSAM administration. Unenhanced low dose computed tomography was acquired at 128 kV and 17 mAs and reconstructed with 5 mm slice thickness. For SPECT 128 frames (25 s/frame) were collected with a 128 × 128 matrix and attenuation and scatter slices were reconstructed iteratively. The fusion images were computed via e.soft 2007 application package (Siemens Healthcare, Erlangen, Germany). In the subsequent qualitative visual analysis any noticeable tracer accumulation in the anterior abdominal wall or in the falciform ligament was registered. In cases with an apparent tracer uptake in these locations strategies to reduce the risk for non-targeted 90-yttrium diversion to the abdominal wall were evaluated.

### Selective internal radiation therapy using 90-yttrium (SIRT)

90-yttrium administration (TheraSphere™, BTG, Hertfordshire, United Kingdom) was performed on an average of 45 days after the initial DSA (range: 16 – 86 days). The therapeutic DSA was performed in the same way as the pretherapeutic DSA using the same equipment. Patients whose HFA could primarily not be occluded but who did not present with any ^99m^Tc-HSAM accumulation in the anterior abdominal wall or in the falciform ligament, received SIRT without any special precautions. In patients, whose HFA could primarily not be coil embolized and who presented with a ^99m^Tc-HSAM accumulation in the anterior abdominal wall or in the falciform ligament, a reattempt of probing and transient occlusion of the HFA with gelfoam (Gelitaspon, Gelita Medical, Amsterdam, The Netherlands) was performed. If probing and occlusion of the HFA was not feasible, ice packs were administered on the ventral mid abdominal wall prior to 90-yttrium administration.

### Follow up

All patients stayed for 48 hours after 90-yttrium administration as inpatients and were monitored for any adverse event especially acute radiation dermatitis, periumbilical skin rash, or abdominal pain. Furthermore, the patients had been instructed to contact the hospital in case of any adverse event after their discharge.

## Results

In 19 of 606 patients (3%) a patent HFA was detected by DSA; however in 48 patients (8%) a slight ^99m^Tc-HSAM accumulation in the ventral abdominal wall was found but no HFA was detected. In 18 of the 19 patients with angiographically patent HFA it arose from a branch of the left hepatic artery. Only in one patient it originated from a branch of the right hepatic artery.

**Figure 1:**
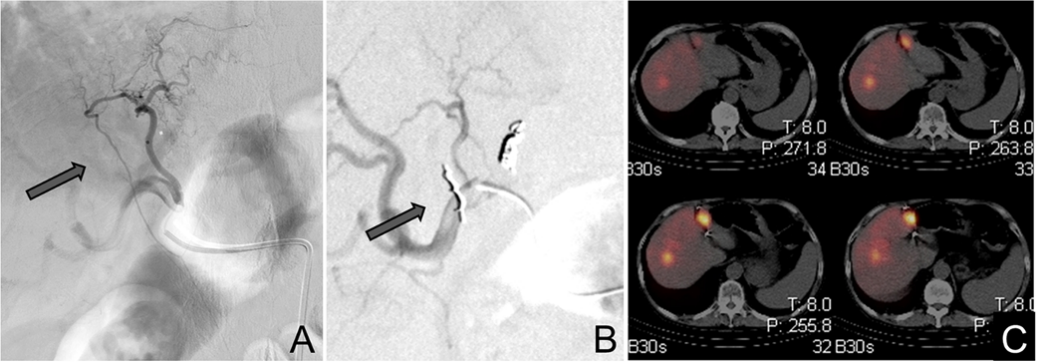
**Example of a coilable hepatic falciform artery (HFA).** Primarily patent HFA visible in the pretherapeutic DSA **(A)** which was successfully coil embolized **(B)**. Afterwards no abdominal wall ^99m^Tc-HSAM acculumulation was detected in the SPECT/CT **(C)**.

**Figure 2:**
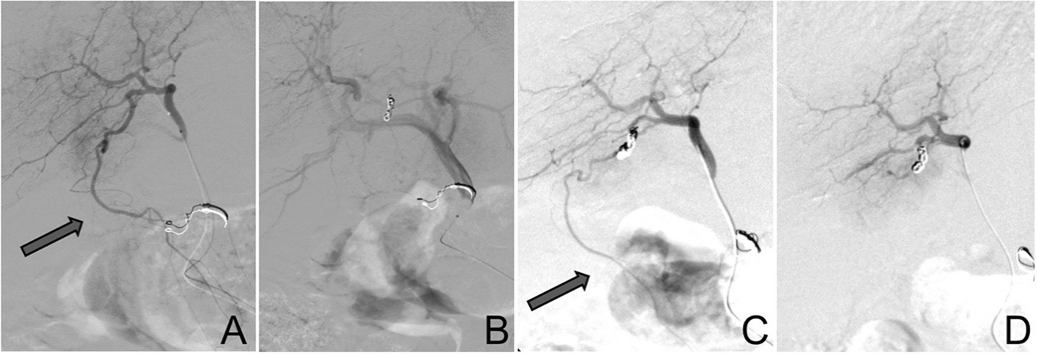
**Example of a coilable, but subsequently partially recanalized hepatic falciform artery (HFA).** Patent HFA visible in the DSA **(A)** which initially was coil embolized **(B)** but which proofed partially recanalized in the therapeutic DSA **(C)**. Subsequently it was reoccluded using gelfoam **(D)** prior to the 90-yttrium administration.

Eight of these 19 patients did not receive 90-yttrium in the hepatic vessel bed containing the HFA due to clinical deterioration (n = 4), persistent duodenal shunting (n = 1), insufficient tumor ^99m^Tc-HSAM accumulation (n = 1), or because the liver tumor was located exclusively in the contralateral liver lobe (n = 2). Hence, only 11 patients received 90-yttrium in the hepatic artery from which the HFA arose. In three of these 11 patients the HFA could initially be coil embolized. None of these patients showed any ^99m^Tc-HSAM accumulation in the ventral abdominal wall or the falciform ligament (Figure 1A-C). In two of these three patients a partial reopening of the HFA was discovered during subsequent therapeutic DSA. Thus, the HFA was reoccluded with gelfoam prior to the 90-yttrium administration (Figure 2A-D). All three patients tolerated SIRT well without any complications.

**Figure 3:**
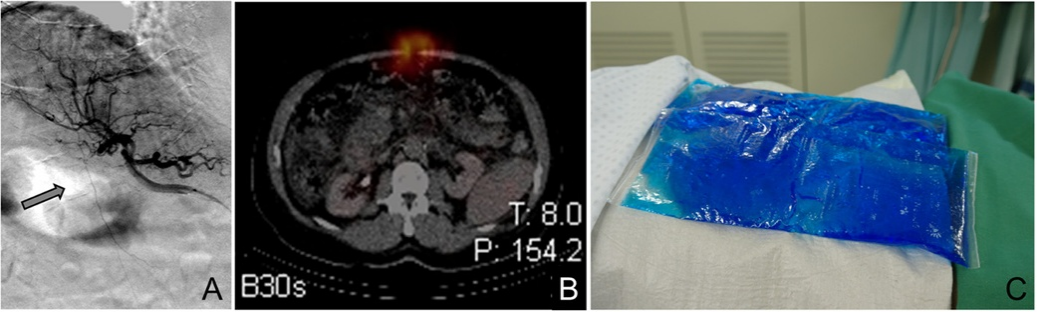
**Example of a not coilable hepatic falciform artery (HFA).** Patent HFA detectable in DSA **(A)** which could not be coil embolized. In the SPECT/CT an abdominal wall ^99m^Tc-HSAM acculumulation was detected **(B)**, hence to prevent SIRT sequela ice packs were placed on the abdominal wall prior to 90-yttrium administration **(C)** which was finally tolerated well.

In eight out of these 11 patients the HFA could not be occluded primarily. Four of these eight patients did neither exhibit any ^99m^Tc-HSAM accumulation in the anterior abdominal wall nor in the falciform ligament in subsequent SPECT/CT. Consequently, the flow in the HFA was regarded as non-significant and therefore these four patients received SIRT without any special precautions. All four patients tolerated SIRT well. The remaining four patients with a persistently patent HFA presented with a ^99m^Tc-HSAM uptake in the anterior abdominal wall (n = 3) or in the falciform ligament (n = 1). Hence, a reattempt of HFA occlusion with gelfoam was performed in all four patients, but succeeded only in two of them. These two patients received SIRT without any complications. In the remaining two patients the HFA was persistently not probable, so ice packs were placed on the anterior mid abdominal wall of these patients during 90-yttrium administration (Figure 3A-C) aiming on vasoconstriction of the cutaneous vessels and a consecutive flow reduction in the HFA. Both patients received SIRT and tolerated it well.

## Discussion

This retrospective study points out two main messages: First, an angiographically patent HFA is only detected in a small percentage (3%) of patients undergoing SIRT. Secondly, according to our experience, a patent HFA is not a contraindication for SIRT, especially if certain precautions such as coil embolization of the HFA or ice pack administration on the ventral mid abdominal wall are carried out.

Inadvertent distribution of 90-yttrium to the ventral abdominal wall via a patent HFA has been described as a complication of SIRT which may result in acute radiation dermatitis, periumbilical skin rash, or severe mid abdominal pain (Leong et al. 2009; Bhalani and Lewandowski 2011). For protective reasons, in cases of a patent HFA a pretherapeutic occlusion of the HFA or a superselective SIRT sparing the HFA dispensing vessel have been proposed (Bhalani and Lewandowski 2011; Kao et al. 2011; Haggerty et al. 2012). But recently, Wang et al. ( 2013) reported on radioembolization in five patients with a patent HFA. Like in our two patients with persistently not probable HFA, Wang et al. did not occlude the HFA but solely placed ice packs on the ventral mid abdominal wall and experienced no complications in all five patients.

In the current cohort, a patent HFA was found only in 3% of all patients. This is in accordance with results of a large study cohort in which HFA was seen in 2% of 1250 patients (Baba et al. 2000). An even smaller prevalence of an angiographically patent HFA (0.5%) was reported by Ahmadzadehfar et al. ( 2011) who investigated 192 patients. They found a ^99m^Tc-HSAM accumulation in the anterior abdominal wall in 9% of patients, which is comparable to the currently reported 8%. Ahmadzadehfar et al. occluded the sole angiographically detected patent HFA. In the remaining patients with ^99m^Tc-HSAM accumulation in the anterior abdominal wall they performed radioembolization without any modifications in the treatment plan regarding the HFA and reported self-limiting abdominal muscle pain in only one patient as a possible symptom of a radiation induced complication. However, although only minor adverse events were reported in this study, we suggest protective procedures such as embolization of the HFA or ice pack administration on the ventral abdominal wall prior to 90-yttrium administration. Thus, the (mainly small) risk of side effects due to microsphere diversion to the abdominal wall may be reduced.

In a study from Burgmans et al. ( 2012) 12% of 42 patients exhibited a patent HFA in DSA. Interestingly the HFA could be identified in 52% of their patients by computed tomography hepatic arteriography (CTHA) with transcatheteral contrast agent administration in the hepatic arteries. The visualization of the HFA in DSA is affected not only by the size of the vessel but also by balancing of blood pressure between the HFA and the internal mammary artery. The reported high HFA detection rate in CTHA may be due to the common diminutive size of the HFA that leads to just a remote blood flow in the vessel resulting in a higher HFA detection rate in the more sensitive CTHA compared to conventional DSA. However, the implications for the clinical routine are questionable. A direct coil embolization in these minute vessels seems to be not always feasible as their study showed as well (Burgmans et al. 2012). A coil embolization was possible only in two of 22 patients with patent HFA detected by CTHA. The remaining 20 patients received SIRT without further precautions and SIRT was tolerated well by all. This leads to the conclusion that the higher sensitivity of CTHA does not modify the clinical practice because a diminutive HFA with a negligible blood flow is probably not prone to cause SIRT complications. Only the presence of a HFA with a larger diameter, which can be detected by DSA or SPECT/CT, should lead to clinical consequences as discussed above.

Despite the high number of SIRT procedures performed at our hospital between October 2006 and December 2012 we have never experienced acute radiation dermatitis, periumbilical rash, or midabdominal pain due to unintentional distribution of 90-yttrium via a patent HFA. To reduce all potential risks we prophylactically embolized the HFA if possible. Nevertheless, patients whose HFA could not be occluded never experienced any SIRT complications. Particularly, in patients with a patent HFA but without any ^99m^Tc-HSAM accumulation in the anterior abdominal wall SIRT is safe. But even in subjects with ^99m^Tc-HSAM uptake in the anterior abdominal wall and a non coilable HFA SIRT seems to be feasible. In these patients we suggest administration of ice packs on the anterior abdominal wall aiming for a temperature induced vessel constriction and a consecutively reduced HFA flow.

Our study is not without limitations. First and foremost, the diagnostic DSA were performed by different interventional radiologists. However, the procedures to prepare for SIRT are highly standardized at our institution and all images were reanalyzed for this study. Secondly, this was a retrospective analysis. Of course it would be favorable to perform a prospective trial comparing the outcome of two patient groups with and without safety precautions prior to SIRT. However, due to the infrequence of a patent HFA this would have been challenging.

Overall, our data underline the low complication rate of SIRT if specific precautions are considered. Therefore a patent HFA should not be regarded as an absolute or even relative contraindication for SIRT.

## Abbreviations

CTHA: Computed tomography hepatic arteriography
DSA: Digital subtraction angiography
HFA: Hepatic falciform artery
SIRT: Selective internal radiation therapy
SPECT/CT: Single-photon emission computed tomography/computed tomography
^99m^Tc-HSAM: 99m-technetium labelled human serum albumin microspheres.
